# Diffraction-limited storage-ring vacuum technology

**DOI:** 10.1107/S1600577514010480

**Published:** 2014-08-27

**Authors:** Eshraq Al-Dmour, Jonny Ahlback, Dieter Einfeld, Pedro Fernandes Tavares, Marek Grabski

**Affiliations:** a MAX IV Laboratory, Lund University, PO Box 118, SE-221 00 Lund, Sweden

**Keywords:** vacuum, NEG coating, DLSR, copper vacuum chambers

## Abstract

Some of the characteristics of recent ultralow-emittance storage-ring designs and possibly future diffraction-limited storage rings are a compact lattice combined with small magnet apertures. Such requirements present a challenge for the design and performance of the vacuum system. The vacuum system should provide the required vacuum pressure for machine operation and be able to handle the heat load from synchrotron radiation. Small magnet apertures result in the conductance of the chamber being low, and lumped pumps are ineffective. One way to provide the required vacuum level is by distributed pumping, which can be realised by the use of a non-evaporable getter (NEG) coating of the chamber walls. It may not be possible to use crotch absorbers to absorb the heat from the synchrotron radiation because an antechamber is difficult to realise with such a compact lattice. To solve this, the chamber walls can work as distributed absorbers if they are made of a material with good thermal conductivity, and distributed cooling is used at the location where the synchrotron radiation hits the wall. The vacuum system of the 3 GeV storage ring of MAX IV is used as an example of possible solutions for vacuum technologies for diffraction-limited storage rings.

## Abstract 2

A compact lattice and small magnet apertures will be the main characteristics of future diffraction-limited storage rings, adding difficulties for the design of the vacuum system of these machines. The use of NEG coatings and distributed absorbers could provide a solution to overcome these challenges.

## 1. Introduction   

The new generation of synchrotron light sources is designed to have an electron beam that has a very small emittance, which allows the photon beam to approach the diffraction limit. As the emittance of the electron beam is related inversely to the third power of the number of magnets and in order to accomplish a low-emittance electron beam, the storage ring should be designed to accommodate a large number of magnets; apparently, this would increase the circumference of the machine and accordingly the construction costs. One way to overcome this is to try to reduce the size of the magnet units while keeping their number as large as possible, and to have a compact lattice. This has an impact on the vacuum system, as the conductance of the vacuum chamber becomes smaller (the conductance of a long circular tube is directly proportional to the third power of the tube diameter) making it difficult to provide the required pumping down. Moreover, the small apertures in the magnets make it more challenging for the vacuum system to manage the synchrotron radiation (SR) power deposited on the chamber walls.

## 2. MAX IV 3 GeV storage-ring layout   

The MAX IV 3 GeV storage ring is an example of a possible approach to the vacuum system design to manage the limitations from the lattice and magnets design of a low-emittance storage ring; an approach that could possibly be adapted for a future diffraction-limited storage ring (DLSR) (Tavares *et al.*, 2014 ▶).

The MAX IV facility is under construction in Lund, Sweden, and will have two storage rings, with 3 GeV and 1.5 GeV beam energy. The 3 GeV storage ring has a circumference of 528 m and will have a multi-band achromat lattice; the beam will have a bare lattice emittance of 0.33 nm rad, which reduces to 0.2 nm rad as insertion devices are added.

**Figure 1:**
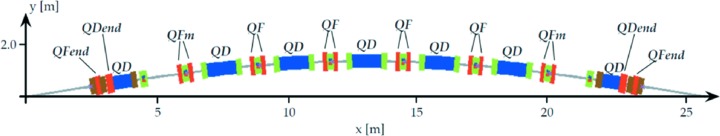
Schematic layout of the 3 GeV storage ring achromat of MAX IV.

The 3 GeV ring has 20 achromats; the schematic of one achromat is shown in Fig. 1 ▶ and consists of seven cells, *i.e.* five unit cells, each with a 3° bending magnet, and two matching cells, with dipoles which have 1.5° deflection angles. Long straight (LS) sections are located at the start of the achromats, and are mainly used for the insertion devices (ID), and each achromat will have two short straight (SS) sections, used to accommodate the main and harmonic RF cavities, as well as a variety of diagnostic equipment and to extract the photon beam to the front-ends (FEs). Each magnet cell is made of one magnet block that houses several magnets (dipole, quadrupoles, sextupoles, octupoles and correctors) (Johansson *et al.*, 2014 ▶). The vacuum system is divided into sections which follow the achromats definition, with sector valves on each side of the LS.

## 3. Vacuum technology for low-emittance storage rings/DLSRs   

The main purpose of the vacuum system of low-emittance DLSRs is to have low dynamic pressure which gives good beam lifetime, and to handle the power deposited by SR. With these two objectives in mind, the vacuum system of MAX IV has been designed taking into consideration the limitations from the magnets and lattice design.

### 3.1. Design of MAX IV vacuum system   

The magnets of the 3 GeV storage ring have a small aperture of diameter 25 mm, making it difficult to have chambers with an antechamber where the photon beam can pass to lumped absorbers, which in turn absorb the power from the SR. As a result, the vacuum chambers within the magnets have a 22 mm inside diameter with 1 mm wall thickness. Distributed cooling channels are welded to the chambers allowing the power deposited by SR to be safely removed. The clearance between the chambers and the magnets poles is 0.5 mm; the chambers are produced with tight mechanical tolerances to avoid any interference with the magnet poles and coils (for example, all tolerances of the chambers should be less than 0.3 mm). In addition, dummies of the chambers were manufactured and provided to the magnet suppliers, and will serve as a tool to verify that the clearance with the magnets is preserved, particularly with the magnet coils, which have looser mechanical fabrication tolerances.

Silver-bearing (0.085%) oxygen-free copper (OFS-C10700) has been chosen as the alloy of the chamber body since it allows the heat to be transmitted efficiently to the distributed absorbers and it is known for having a substantial strength and high resistance to softening due to elevated temperature, which is a good choice for applications where heating cycles could occur.

One beam position monitor (BPM) is located in each cell; in total, ten BPMs are placed between the chambers of each achromat and they are fastened directly onto the magnet blocks. As the chambers absorb the heat from the photon beam, they will expand; accordingly, each vacuum chamber has bellows welded onto its extremities; these will mainly serve to absorb the deformation that occurs due to chamber expansion, reducing the stress on the chamber and avoiding any movement of the BPMs which are located in between, keeping the BPM stable. The BPM blocks are slightly heated up by SR transferred from the neighbouring chambers; however, the symmetrical design of the BPM block allows it to heat up with symmetrical deformation of the body, resulting in only a minor shift of the centre of the block.

Standalone bellows, with a larger stroke than that of the chambers’ bellows, are located on each side of the LS; the larger stroke is needed as the vacuum chambers of the LS section will be baked *in situ*.

Some parts of the vacuum chambers that are made of stainless steel (such as pumping ports and BPM blocks located on each side of the LS) will be used to accommodate the fast orbit correctors (Sjöström *et al.*, 2011 ▶).

Stainless steel ribs will be welded to the vacuum chamber body; they will be used for holding the vacuum chamber during bakeout and NEG coating and also for supporting the chambers inside the magnets.

**Figure 2:**
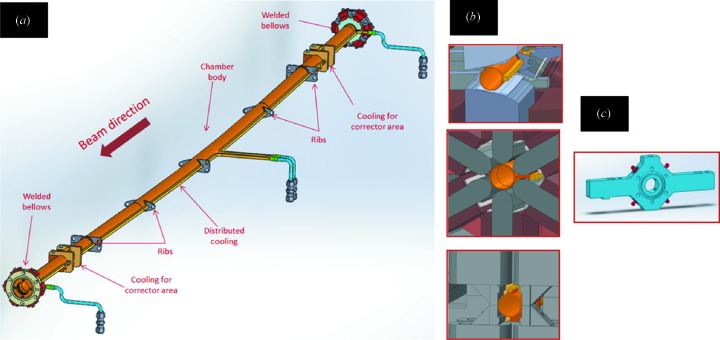
(*a*) Layout and main parts of one vacuum chamber of the MAX IV 3 GeV storage ring. (*b*) Cross sections of the chambers inside the dipole, sextupole and corrector magnets. (*c*) The BPM block.

Fig. 2 ▶ shows the layout and the parts of one vacuum chamber, cross sections inside the magnets and the BPM block of MAX IV.

**Figure 3:**
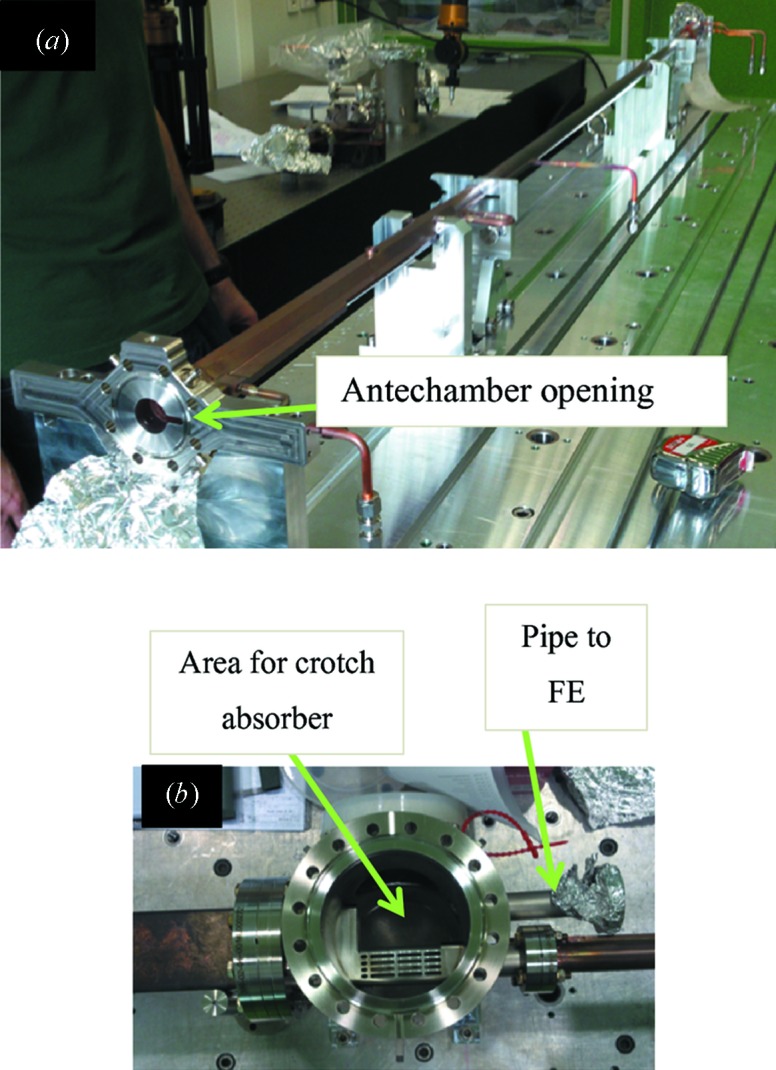
(*a*) VC1: the first chamber after the LS, with opening (antechamber) for SR. (*b*) Crotch absorber chamber.

The photon beam extraction occurs in the first SS section of the achromat; a crotch absorber located inside a stainless steel chamber will allow the photon beam to pass, followed by a cooled absorber that permits around ±1 mrad in the horizontal and ±0.48 mrad in the vertical of ID radiation to pass. Fig. 3 ▶ shows some of the photon beam extraction chambers.

Finite-element analyses (FEAs) were performed to guarantee the thermal and mechanical stabilities of the vacuum chambers, absorbers, RF fingers, BPM, *etc*. As an example, FEA was performed to estimate the stress, deformation and temperature distribution on the vacuum chambers due to SR; the results were within the design criteria.

**Figure 4:**
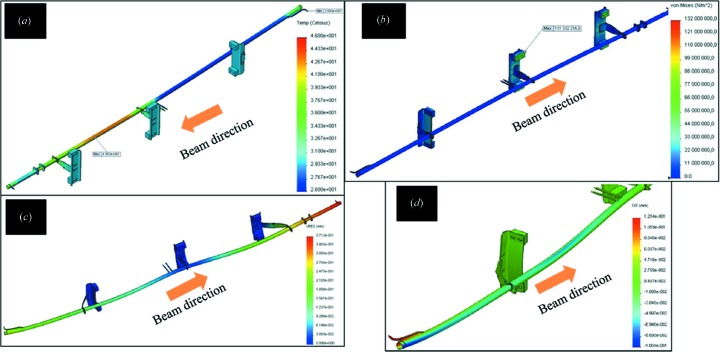
FEA results for one vacuum chamber: (*a*) temperature distribution (°C), (*b*) stress (MPa), (*c*) overall deformation (mm), (*d*) radial deformation (mm).

Fig. 4 ▶ shows the thermal and the structural results of the FEA for VC-6 (the vacuum chamber located in the unit cell); the maximum power density from the bending-magnet radiation was 9.4 W mm^−2^. The results show that the maximum temperature is 319 K, which is located close to the area of the highest power density (end of the dipole). The maximum stress is 132 MPa, which is on the stainless support, while on the chamber body (copper) the maximum stress is 12 MPa. The chamber will elongate by 0.37 mm and the bellows on each side will absorb this elongation. The maximum radial deformation is 0.12 mm, located at the extremities of the chamber, where the bellows will absorb this. The maximum radial deformation on the chamber body is less; this means that the clearance to the magnets is preserved.

As the copper chambers have a thin wall of thickness 1 mm, conservative design criteria have been chosen, where the maximum thermal stress should be less than 50 MPa.

The total power from SR is proportional to the beam current and the fourth power of the beam energy, and inversely proportional to the bending radius of the dipole magnet (Gröbner *et al.*, 1983 ▶), while the power density is proportional to the fifth power of the beam energy. For MAX IV, with a beam current of 500 mA, the maximum power density on the vacuum chambers is 9.4 W mm^−2^; accordingly MAX IV was able to use the chamber body as an absorber of the SR power. This was possible as the maximum stresses and temperatures due to this power were within the design criteria for OFS copper. For other machines, this might not be possible. In cases where the power and the power densities are higher [for example, the ESRF upgrade project (Farvacque *et al.*, 2013 ▶)], other design philosophies need to be used; it might be necessary to change the material used at the area where the SR bombards the chamber to an alloy that can withstand higher stress (*e.g.* Glidcop), and/or to change the cooling design. If the power is still too high to handle, then a design with an antechamber where the power is absorbed using lumped absorbers could be adapted. Clearly, this needs to be considered in the design of the lattice and magnets. The chamber design is more complicated, adding difficulties for their production.

### 3.2. NEG coating   

The conductance of the vacuum chambers of DLSRs is very low due to the small magnet aperture. Lumped pumping would not be as efficient in reducing the pressure to the required level, and, with a compact lattice, it would be difficult to have enough space to place the pumps. Distributed pumping is the obvious solution to solve the conductance limitation.

One way to provide distributed pumping is to use NEG strips; nevertheless, they would need space on the antechamber side, which could be difficult to provide (considering the small magnet apertures and the magnet design).

NEG coating is well known for its properties in reducing the photo-stimulated desorption yield and in pumping down gases after activation (Chiggiato & Kersevan, 2001 ▶). It has been used in storage rings for several years, mainly for coating the chambers of narrow-gap insertion devices, which have very small apertures. It has also been demonstrated that NEG coating can withstand at least 30 activation cycles, without significant reduction of its performance (Mazzolini, 2006 ▶).

Synchrotron SOLEIL in France extended its use for the chambers inside the magnets, with more than 50% of the SOLEIL storage ring vacuum chambers being NEG coated; in addition, conventional pumping was connected to the SOLEIL coated chambers (Herbeaux *et al.*, 2008 ▶). MAX II extended the use of NEG coating to the dipole chambers, with the results showing good performance (Hansson *et al.*, 2010 ▶).

The entire circumference of the MAX IV storage ring will be NEG coated (with the exception of the pulsed magnets, a few diagnostic components and the RF cavities); in general, only three ion pumps will be used per achromat. As MAX IV will be the first storage ring which will use NEG coating extensively, research and development (R&D) was needed. The main purpose of this R&D was to assure the possibility of the use of NEG coating for small-aperture bent tubes and for coating the antechamber of the photon extraction vacuum chambers; it was also to make sure that all production processes are safe for the NEG coating performance. A collaboration between CERN and MAX IV Laboratory has been set up to investigate these issues (Calatroni *et al.*, 2013 ▶).

#### 3.2.1. Surface preparation and cleaning   

To ensure that NEG coating will have good adhesion to the copper chambers, all the raw tubes should have surface treatment. The treatment consists of three steps: degreasing, followed by etching, and finally passivation. The etching will remove around 50 µm from the metal, leaving a clean surface for NEG coating.

After the surface treatment, the tubes will follow several manufacturing processes, where the chambers could be exposed to contamination; accordingly, final cleaning should be performed before NEG coating. The results of the cleaning process should fulfil the standards established by CERN for compatibility with NEG coating. X-ray photoelectron spectroscopy (XPS) will be used on cleaning samples to assure this.

#### 3.2.2. NEG coating for small-aperture bent tubes   

**Figure 5:**
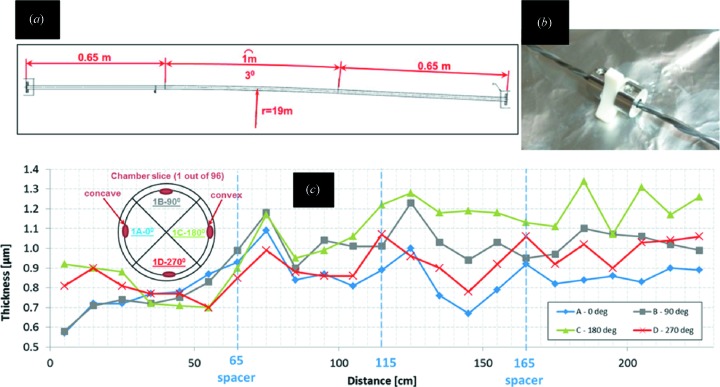
(*a*) Layout of the test bent vacuum chamber for NEG coating. (*b*) The spacers used to hold the cathode. (*c*) XRF results for the coating thickness at several intervals of chamber length and at different positions.

The MAX IV achromat chambers are around 2.5 m long, 1 m of which is bent with a bending radius of 19.1 m. To be able to coat the bent part without having the cathode touching the tube, three ceramic spacers were placed at the start, middle and end of the bent part. Following the coating, the coating thickness was measured using the X-ray fluorescence (XRF) technique at several positions. The tube was sliced every 10 cm and each slice was cut radially into four pieces. The result of the XRF thickness measurement is presented in Fig. 5 ▶; the thickness is within the established limits (0.5–2 µm).

#### 3.2.3. NEG coating for the antechamber   

**Figure 6:**
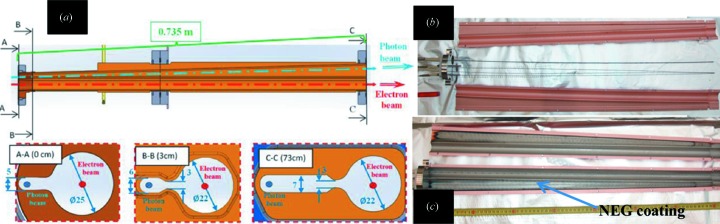
(*a*) Layout of the chamber with antechamber for the MAX IV ring. (*b*) Test piece of the chamber before coating. (*c*) Test piece of the chamber after coating (grey).

The coating of the antechamber is a challenge as the aperture is small; for MAX IV it varies from 5 mm to 7 mm (see Fig. 6 ▶). Testing was carried out at CERN with several coating settings (magnetic field, number of cathodes, discharge gas pressure, *etc.*) to find the best conditions for coating this type of chamber.

#### 3.2.4. Verification of manufacturing process and possible effect on NEG coating   

The MAX IV chambers require the use of several manufacturing techniques before a chamber is ready for use; all these techniques should not affect the NEG coating performance. For example, to achieve the antechamber shape, the electrical discharge machining technique is used, which uses Zn cutting wire. Investigations have been made to verify whether this has any effect on the NEG coating performance. For this, a test chamber was manufactured using this technique and then coated; activation cycles were performed, with the results showing no evidence of NEG coating peel-off. Also, an activation test of a sample made with this technique using XPS showed no effect on the NEG coating performance.

### 3.3. Installation   

**Figure 7:**
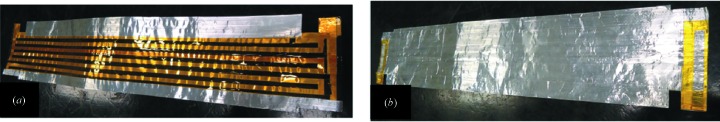
The heating tapes which will be used for the *in situ* bakeout of the Sirius vacuum chambers, (*a*) without the aluminium layer, (*b*) with the aluminium layer.

The NEG-coated chambers should be heated up to activate the NEG; no venting can be performed after this. Therefore, the installation procedure should consider this. *In situ* bakeout where the chamber is covered with heaters and baked while inside the magnets is the best solution, as this reduces the manipulation of the chambers and also reduces the installation time. However, there should be sufficient space to place the heaters and insulation between the magnets and the chambers; in addition, and as the chamber will expand, bellows with sufficient stroke should be placed within the vacuum chambers to absorber this deformation without adding stresses to the chambers. The Sirius project (which has a similar design concept as MAX IV) will use thin polyimide heating tape wrapped around the chambers for activation *in situ*; it has a thickness of 0.4 mm and has an aluminium layer which will shield the heat from the magnets and reduce the power needed for activation (see Fig. 7 ▶) (Sirius, 2013 ▶). In the case of MAX IV, and owing to its compact lattice, it was not possible to place bellows with large stroke to absorb the elongation of the chamber during the activation. Accordingly, each achromat will be assembled on assembly tables (which have similar positioning tolerances of the BPM blocks as those on the magnets), then baked in an oven while hanging from a strong-back which will allow the chambers to expand freely; after this, the achromat will be placed inside the open magnets. The handling of the chambers is critical, but with proper design and FEA of the strong-back to be used and testing, this could be solved. *In situ* bakeout will be carried out for the LS and SS sections which accommodate the RF cavities. In addition, MAX IV is planning to have a fully assembled and activated spare achromat, which will serve as a replacement for any achromat in the machine if needed.

## 4. Conclusion   

The vacuum system design, manufacturing and installation of future DLSRs are challenging. The MAX IV and Sirius projects are examples of recent ultralow-emittance storage-ring designs which make use of new techniques and improve known methods. The forthcoming commissioning of these machines will allow an important validation of such procedures and techniques for the construction of this new type of storage ring.

Handling the power from SR when there is only a small space to place absorbers is a challenge. This was made possible for MAX IV and Sirius due to the relatively low power densities on the chambers.

The extensive use of NEG coating is likely to be the future technique for pumping down and reducing the dynamic outgassing of this type of storage ring. Research was needed to solve technical issues in adapting this technique for the coating of the chambers.

The installation of the chambers while considering the need of NEG activation is an important issue which should be carefully studied; FEA and testing of the items to be used during the installation is essential.

Finally, it cannot be overemphasized that the vacuum system requirements need to be considered during the early stages of the lattice and magnets design. This could allow the use of simplified solutions for the design, manufacturing and installation of the vacuum system.
